# Sperm metabolism is altered during storage by female insects: evidence from two-photon autofluorescence lifetime measurements in bedbugs

**DOI:** 10.1098/rsif.2015.0609

**Published:** 2015-09-06

**Authors:** Klaus Reinhardt, Hans Georg Breunig, Aisada Uchugonova, Karsten König

**Affiliations:** 1 Department of Animal Evolutionary Ecology, University of Tuebingen, Auf der Morgenstelle 28, 27076 Tuebingen, Germany; 2 Department of Animal and Plant Sciences, University of Sheffield, Sheffield S10 2TN, UK; 3 Applied Zoology, Department of Biology, Technische Universität Dresden, 01062 Dresden, Germany; 4 Department of Biophotonics and Laser Technology, Saarland University, Campus A5.1, 66123 Saarbrücken, Germany; 5 Jenlab GmbH, Schillerstrasse 1, 07745 Jena, and Science Park 2, Campus D1.2, 66123 Saarbrücken, Germany

**Keywords:** biophotonics, sexual selection, sperm competition, cell ageing, sperm metabolism

## Abstract

We explore the possibility of characterizing sperm cells without the need to stain them using spectral and fluorescence lifetime analyses after multi-photon excitation in an insect model. The autofluorescence emission spectrum of sperm of the common bedbug, *Cimex lectularius*, was consistent with the presence of flavins and NAD(P)H. The mean fluorescence lifetimes showed smaller variation in sperm extracted from the male (tau m, *τ*_m_ = 1.54–1.84 ns) than in that extracted from the female sperm storage organ (tau m, *τ*_m_ = 1.26–2.00 ns). The fluorescence lifetime histograms revealed four peaks. These peaks (0.18, 0.92, 2.50 and 3.80 ns) suggest the presence of NAD(P)H and flavins and show that sperm metabolism can be characterized using fluorescence lifetime imaging. The difference in fluorescence lifetime variation between the sexes is consistent with the notion that female animals alter the metabolism of sperm cells during storage. It is not consistent, however, with the idea that sperm metabolism represents a sexually selected character that provides females with information about the male genotype.

## 1. Introduction

Fluorescence lifetime studies have been used to non-invasively assess the metabolic state of cells by examining the redox ratio of the cell. The redox ratio, the proportion of NAD(P)+ over NAD(P)H, represents the relative proportions of glycolysis and oxidative phosphorylation in a cell. This procedure has, for example, been successfully employed to identify stem cells compared with differentiated cells, normal cells compared with cancer cells and sperm cells in different compartments [1–10]. Some concerns have arisen over the use of monoexponential fluorescence decay models in some of these applications, because the presence of several redox-related autofluorescent molecules, such as free and protein-bound NAD(P)H, as well as free and protein-bound flavins, would require bi- or triexponential decay models [11]. Because flavins and NAD(P)H vary in their fluorescence excitation and emission spectra, it is desirable to augment the lifetime analyses in metabolic mapping with examinations of spectral properties.

Animal sperm cells may be a particularly interesting target in which to compare lifetime analyses and metabolic mapping, for at least three reasons. (i) Sperm dysfunction has been suggested to be the single most important factor of known aetiology to cause infertility in humans [12]. Many molecular mechanisms that disturb sperm function involve aspects of cell metabolism and the action of oxygen radicals [13]. Animal sperm cells may be an easily assessable model with which to pilot fluorescence lifetime technology in order to examine aspects of cell metabolism and oxygen radical production. (ii) Sperm cells of several species can switch between the oxidative phosphorylation and glycolysis pathways. However, for most species the overall concentration, or even the presence, of flavins and NAD(P)H is unknown. (iii) Variation in sperm characters is large across species, as well as within species—a striking pattern that requires an evolutionary explanation but has been largely restricted to sperm morphology [14]. It has been suggested that much of the variation in sperm cells is shaped by sexual selection taking place after mating (postcopulatory sexual selection, PSS). This hypothesis postulates that sperm cells reflect the genetic quality of the male. Selection for sperm cells that are the most successful in fertilizing the eggs will represent selection for male genotypes producing these sperm [14–16].

Selection at the level of the sperm cells happens if sperm of one male is superior to sperm of other males within the same female, a concept called sperm competition [14–16], or if females select the sperm of specific males only (called cryptic female choice [17]; if exclusively based on the characteristics of this male's sperm: sperm choice). Competition among sperm implies that sperm metabolism is involved to at least some degree in the contest between sperm on their way to fertilization. The idea that evolution proceeds via these forms of PSS has empirical support. For example, the success of sperm competition varies between males and within females [18], and sperm characters and females were experimentally shown to co-evolve [19]. However, in PSS, it is not known how exactly sperm from different males outcompete each other, or what characteristics females could use to select specific sperm genotypes. While, in principle, sperm metabolism may carry information about the producing male (i.e. be related to a male's genotype), a number of objections exist for this suggestion. First, sperm cells age over the course of their cellular lifetime and thereby show altered metabolic activity (reviewed in [20]) and fertilization success [20–22]. If the metabolic activity is closely correlated to the age of sperm cells, it will provide only limited information about the genotype of the producing male. Second, the metabolic rate and oxygen radical production of sperm extracted from the male did not predict sperm metabolism if sperm of that same male were examined after sampling from the female [9]. Selecting male genotypes based on the oxidative metabolism of their sperm is, therefore, difficult. Third, ejaculated sperm can be stored alive for extended periods inside the female reproductive tract across many animal species. While the longest recorded post-ejaculation lifespan is 30 years in some ants [23], sperm of other insects, reptiles, decapods and bats are also known to be especially long-lived in the female tract [24–27]. The occurrence of extended sperm storage durations in diverse taxa suggests that one or more female mechanisms to delay the ageing of the sperm cell have evolved repeatedly and are partly based on similar mechanisms [27]. One way to achieve a delay in sperm deterioration during storage would be for females to manipulate the cellular metabolism of the stored sperm in such a way as to reduce oxidative damage, a major cause of cellular ageing. In support of this suggestion, the sperm cells of two insect species showed both a reduced metabolic rate and a reduced production rate of intrasperm oxygen radicals during sperm storage within the female, but not within the male [9,10]. However, as females interfere with sperm metabolism, the suitability of sperm metabolism as an indicator of the genetic quality of the male is reduced.

**Figure 1.**
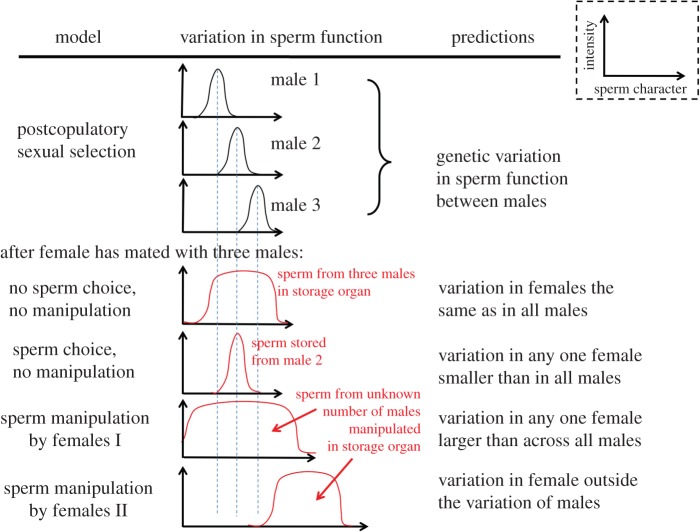
Variation in sperm function in females (red curves, bottom four graphs), compared with that in males (black curves, top three graphs), as expected by neutral variation, by postcopulatory sexual selection (sperm choice) and by two different female manipulation models.

Here, we employ fluorescence lifetime imaging (FLIM) and autofluorescence spectroscopy to (i) assess the presence of flavins and NAD(P)H and (ii) test assumptions of the PSS and the female manipulation hypotheses. We do so in a model system of sexual selection research, the common bedbug, *Cimex lectularius*, by comparing spectral and lifetime properties of the intrinsic fluorescence of sperm cells extracted from the male and the female. We used two evolutionary models to make *a priori* predictions about expected patterns of variation in sperm metabolism. Under the female sperm manipulation hypothesis, the variation in sperm metabolism should be larger in females than across all males, or should fall outside the sperm metabolic variation observed within males (figure 1, bottom panels). By contrast, if females select sperm cells based on metabolic parameters of these cells (an assumption of PSS), the variability in sperm metabolism measured in females should only be a subset of that seen in males (figure 1).

## 2. Material and methods

### 2.1. Study animals

Male and female bedbugs were obtained from a standard culture maintained for several years at the University of Sheffield (UK) [28–30]. Males and females were taken from large mixed-sex laboratory cultures, where they can be found to copulate regularly when kept together [31,32]. Prior to the measurements, females were separated from males for 1–3 days.

### 2.2. Sample preparation

Male storage containers for sperm as well as the female storage organs were dissected just prior to the measurements as described previously [10,32,33]. Sperm were dissected out on a microscopic slide into a drop of phosphate-buffered saline (PBS) and kept in PBS for a few seconds to a few minutes before the measurements. Sperm were not counted, but there were several hundred sperm cells per sample. In samples from both sexes, sperm showed different densities from dense aggregations of several hundred cells to individualized sperm. All measurements were carried out at room temperature.

Solutions of 50 nM MitoTracker green (Invitrogen, M-7514) and 3 µM ethidium bromide (Sigma, E-1510) were applied according to the manufacturers’ instructions.

### 2.3. Two-photon autofluorescence imaging and spectroscopy

Measurements were performed with the multi-photon tomograph DermaInspect (Jenlab GmbH, Jena, Germany) described in more detail elsewhere [5–9]. Briefly, the system consists of a tunable femtosecond (fs) laser source (MaiTai XF1 with a DeepSee unit; Newport/Spectra Physics, Newport, USA), a scan-detector module and beam-steering and high-NA focusing optics. The scan-detector module contains a pair of galvo-scanning mirrors, a beam expander and a dichroic mirror to separate excitation and signal light. The fs laser provides sub-100 fs pulses at a repetition rate of 80 MHz in the tuning range of 710–920 nm with an output power of 0.5–1.1 W depending on the centre wavelength. The focusing optics are used for focusing the laser light onto the sample and for collecting the signal light. To record an image, the laser focus is pixel-wise scanned over the sample area and exclusively excites fluorescence within the focal volume by two-photon absorption. Signal light that reaches the focusing optics is synchronously pixel-wise detected and its intensity displayed in grey-scale images. In the FLIM mode, the arrival time of the signal photons is measured by time-correlated single-photon counting (TCSPC) [34]. The signal arrival time can be used to determine the fluorescence decay times for each pixel. A false-colour representation of the decay times leads to FLIM images. The multi-photon tomography provides optical sections with a maximum field of view of 250 × 250 µm perpendicular to the optical axis at an adjustable depth between 0 and approximately 200 µm. It provides subcellular resolution of about 0.3 µm laterally and 1–2 µm within the axial direction [5]. The temporal resolution used was approximately 200 ps. The fluorescence decays were fitted using commercial fitting software (SPCImage; Becker & Hickl GmbH, Berlin, Germany), taking into account the instrument response function which had been determined previously by recording the second harmonic generation signal from a sample of crystallized urea placed at the focus of the focusing optics. During the measurements, the scanning time for an image of 512 × 512 pixels (256 × 256 pixels for FLIM) was set to 7 s for fluorescence intensity imaging and 13 s for FLIM. The mean laser power incident on the sample was adjusted between 2 and 5 mW. The signals were detected with photomultiplier tubes (for intensity, Hamamatsu H 7724; for TCSPC imaging, PMH-100; Becker & Hickl GmbH). A broadly transparent blue-green colour-glass filter (BG39) was used to protect the detectors from residual laser light in all measurements.

For the spectral measurements, the laser focus was set to a specific location of the sample, and the signals were guided by a fibre in a non-descend geometry to a thermoelectric-cooled charge coupled device-array spectrometer (BTC112; B&W Tek, Newark, DE, USA) and recorded.

The spectrometer provides a wavelength-dependent resolution of a few nanometres and operates in the range of 350–650 nm with a maximum transmission around 525 nm [6,7].

Two-photon images were taken at various excitation wavelengths ranging between 740 and 900 nm with the emission spectra also being recorded. The autofluorescent molecules NAD(P)H and flavins or flavoproteins are excited with 760 nm wavelength, while emitting at 440–470 and 510–530 nm, respectively. If both components are present, intermediate emission peaks are expected at 760 nm excitation or less. Under longer excitation wavelengths, a spectral shift towards longer emission maxima is then expected because of the preferred excitation of flavins. NAD(P)H can no longer be excited at laser wavelengths above 800 nm.

### 2.4. Fluorescence lifetime measurements

The fluorescence decay was modelled as either mono-, bi- or triexponential decay. Build-in statistics were used to compare the goodness of fit of the three decay curves. The deviation between the data points and fitted curves is described by a so-called chi-squared value. The best fit is observed if these chi-squared values are 1 (higher and lower values are possible). In the biexponential decay curves, the average lifetimes (*τ*_m_) were first compared, and later the two lifetime peaks (*τ*_1_) and (*τ*_2_) were inspected separately. *τ*_m_ is calculated as *τ*_m_ = *a*_1_*τ*_1_ + *a*_2_*τ*_2_, and, therefore, takes into account different intensities of the fluorescence component lifetimes. Graphical presentations were also examined using the software package SPCImage (Becker & Hickl, Berlin). The software allows an inspection of the fluorescent molecule's individual lifetime distribution and, therefore, also of whether lifetimes *τ*_1_ and *τ*_2_ consist of one or two peaks.

## 3. Results

### 3.1. Spectral analysis

**Figure 2.**
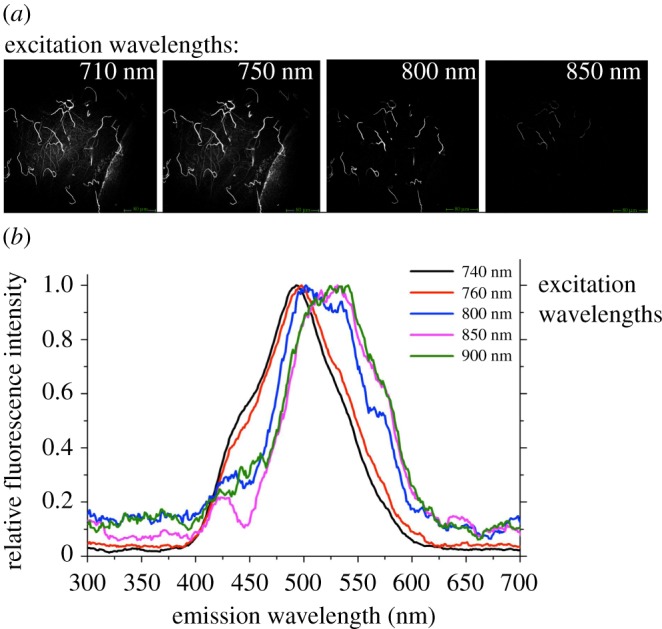
Autofluorescence emission of sperm cells extracted from male bedbugs, *Cimex lectularius*, under different excitation wavelengths: (*a*) multi-photon image examples of four excitation wavelengths, (*b*) shift in emission peaks with longer wavelengths. Data were collected in steps of 1 nm excitation wavelength and averaged across 25 nm steps (i.e. 300–325 nm, 326–350 nm, etc.). The green scale bars in (*a*) represent 80 µm.

Sperm autofluorescence emission peaked at approximately 490–500 nm under 740–800 nm excitation but shifted to approximately 530 nm at excitation with longer wavelengths of 850–900 nm (figure 2).

### 3.2. Fluorescence lifetime analysis

#### 3.2.1. Decay components

**Figure 3.**
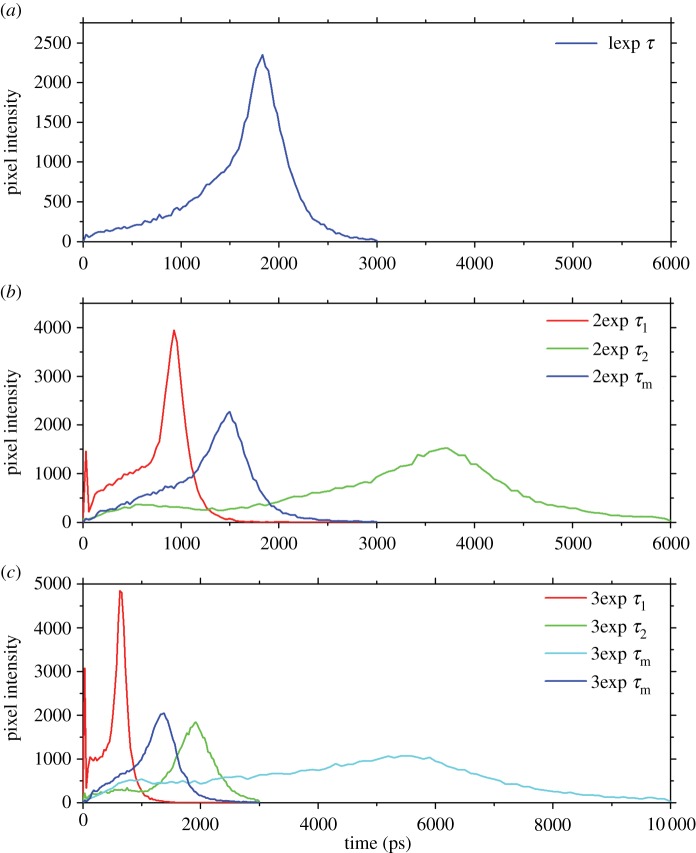
Frequency distribution (histogram) of autofluorescence lifetimes of sperm of common bedbug *Cimex lectularius*, extracted from the male and using different fittings. The monoexponential fit (*a*) assumes a single autofluorescence component; bi- and triexponential fits (*b*,*c*) assume two or three components, respectively (example from male). Excitation wavelength: 760 nm (two-photon).

Figure 3 shows the distribution of fluorescence lifetimes after assumed mono-, bi- and triexponential fitting (*a*–*c*; example from male).

Goodness-of-fit analyses showed a markedly improved model fit of a bi- over monoexponential fit but not from bi- to triexponential fit. Because the goodness-of-fit was not markedly improved by fitting three components, the more parsimonious biexponential fitting was subsequently used.

#### 3.2.2. Average lifetime *τ*_m_ with biexponential decay

**Figure 4.**
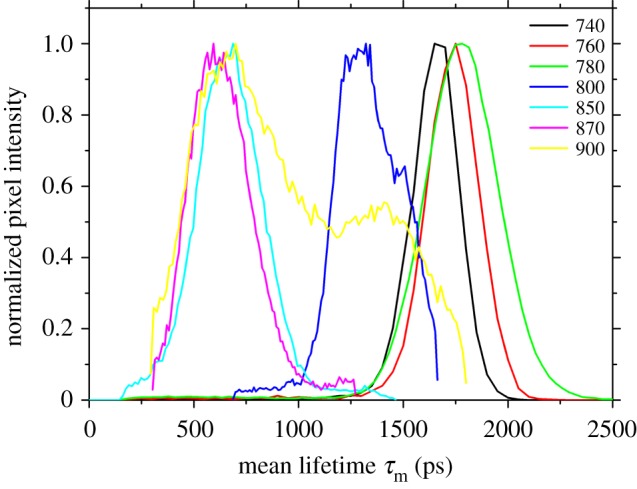
Histograms of fluorescence lifetime decays (*τ*_m_) of sperm cells of bedbugs in relation to variation in excitation wavelength, extracted from the male and examined in a sample of high sperm density.

At excitation wavelengths of 740–780 nm the average lifetime *τ*_m_ peaked around 1700–1750 ps, at wavelengths of 850–900 nm around 700 ps. There was a marked intermediate lifetime peak value at 1400 ps when sperm were excited at 800 nm (figure 4).

**Figure 5.**
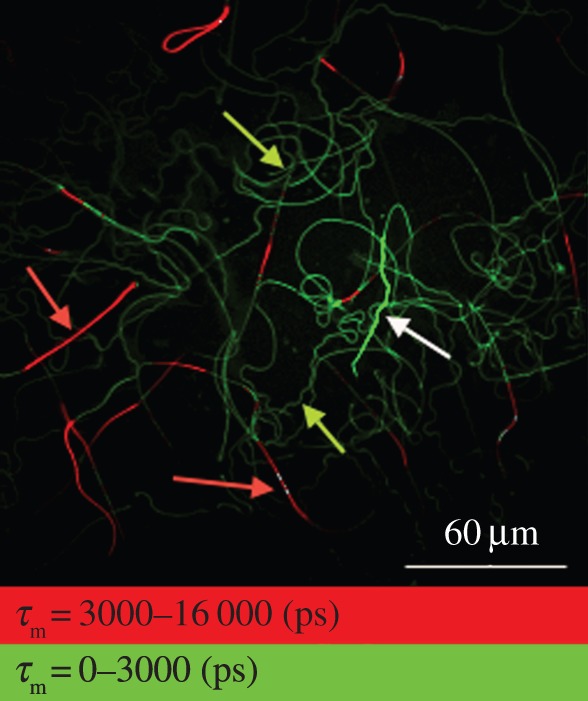
Bedbug sperm cells stained with MitoTracker (green) and counter-stained with ethidium bromide (red). Fluorescence lifetime distribution is shown in two classes, below and above 3 ns, allowing the separation of heads of dead sperm (nuclei with DNA, marked with red arrows), mitochondria in the tails and heads of living sperm (green).

The autofluorescence lifetime may also be combined with lifetime measurements of cellular staining reagents, such as ethidium bromide (lifetime: 5000–6000 ps; Mitotracker: 2000–3000 ps; figure 5), and heads of sperm cells can be distinguished from sperm tails. Specifically, ethidium bromide stains red the nucleic acid in the nucleus of dead sperm cells (figure 5; red arrows), which showed a mean lifetime of *τ*_m_ > 3000 ps. Sperm heads of live cells (not stained with ethidium bromide) appear as a strong green fluorescence (figure 5, white arrow), whereas mitochondria in or around the sperm tails stain faint green (figure 5, green arrows).

### 3.3. Variation between males and females

**Figure 6.**
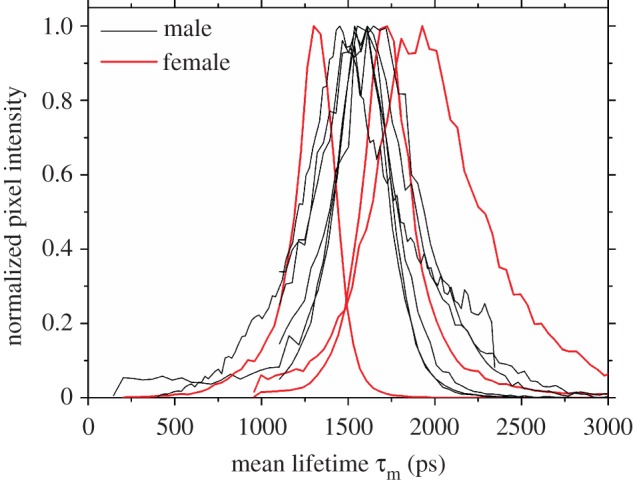
Distribution (histogram) of fluorescence lifetimes for sperm cells of the common bedbug, *Cimex lectularius*. Data for sperm extracted from the female sperm storage organ are shown in red (only the minimum, maximum and mean for females are shown); data for sperm extracted from males are in black. Fluorescence intensity is normalized to facilitate comparability.

Mean lifetimes *τ*_m_ of sperm cells after excitation with 760 nm showed larger variation in sperm cells extracted from females than in sperm cells extracted from males: *τ*_m_ for all male measurements was 1622 ps (range: 1538–1836 ps; standard error: 29 ps = 1.8% of mean; figure 6). In females, however, while the mean of all measurements was similar (1659 ps; figure 6), the variation was much larger (range: 1264–2000 ps; standard error: 44 ps = 2.6% of mean). These data indicate that the sperm metabolic state within the female storage organ is not a subset of the metabolic state observed in males.

Some of the variation between males and females in fluorescence lifetime was caused by large variation between multiple samples from the same individuals. The difference between mean sperm lifetime peaks in individual females from which at least two sperm samples were measured independently (*n* = 6 females) was 276 ps. The respective difference in males with at least two measurements (*n* = 2) was only 119 ps. All six differences found in females were larger than the two differences found in males. This small number is not amenable to statistical analysis. However, we calculated the probability that by chance the two lowest of eight samples belong to one sex, and the six highest to the other. The probability is 0.035 (i.e. two-eighths multiplied by one-seventh) and hence below the *p* < 0.05 probability commonly used in the biological sciences. These data suggest that at least one aspect of sperm function related to *τ*_m_ varies more within females than within males; again, this is not a subset of that observed in males.

**Figure 7.**
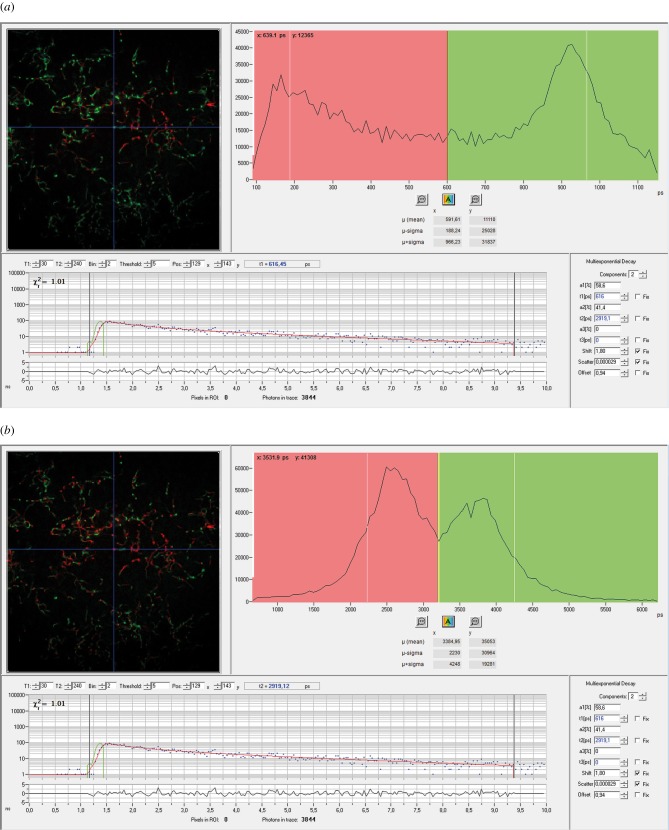
(*a*,*b*) Fluorescence lifetime peaks in two autofluorescent components of bedbug sperm after fitting a biexponential fluorescence decay. Both *τ*_1_ and *τ*_2_ components consist of two peaks each.

The variation in *τ*_m_ across females was examined in more detail by separating the lifetime components under a biexponential decay model at 760 nm excitation. Both components of the biexponential decay, *τ*_1_ and *τ*_2_, showed two peaks each (figure 7). For *τ*_1_, one peak occurred around 180 ps (peak A), and one around 920 ps (peak B; figure 7). For *τ*_2_ these peaks were around 2500 ps (peak C) and 3800 ps (peak D; figure 7).

Peak D was present and pronounced in all three male samples but in less than half of the female samples (six out of 13). If the peak was present in females, it was smaller and often shifted towards longer lifetimes of 3000 ps, sometimes reaching up to 4500 ps (*n* = 3 females).

## 4. Discussion

Insect cells have previously served as model systems in FLIM technology, for example when addressing questions related to chloride transport [35,36] or to the molecular interactions between pathogenic viruses and the host cell [37]. Based on the results presented here we, first, advocate FLIM as a method to investigate sperm metabolism, because the method is easily applicable, non-invasive and there is no need to stain the sperm. The method is readily adaptable to other species and, in fact, has been applied to sperm of a distantly related species, the field cricket *Gryllus bimaculatus* (K Reinhardt, G Breunig, A Uchugonova, K König 2010, unpublished observations). It is noteworthy that even baseline sperm metabolism data are rare for genetic model systems, whereas in other model systems and in humans the relative amount of glycolysis and oxidative phosphorylation in sperm metabolism is often debated [38]. Our method will also be a useful addition to those studies that compare sperm motility under different experimental conditions, or for different species [39].

Second, we demonstrated how this method can contribute to testing an open question in evolutionary biology. We provided evidence that sperm metabolism is altered in the sperm storage organ of female animals, in our case bedbugs, and argued that, therefore, a metabolism-based selection of genetically superior sperm is unlikely. We discuss both aspects below.

### 4.1. Sperm autofluorescence and sperm metabolism

Large concentrations of flavins, mainly FAD, in sperm were revealed in a water strider, a species in the same insect order as the bedbug (Heteroptera). After one-photon excitation with 351 and 458 nm, emission peaked around 510 nm [40]. The intense autofluorescence emission at around 530 nm (excitation at 850 and 900 nm (two-photon), figure 2*b*) is characteristic of flavins. However, the shift from 490 to 530 nm with increasing emission wavelength also shows that, unlike in the water strider sperm, additional components were present in bedbug sperm. The additional components very likely include NAD(P)H, because this molecule emits around 450–480 nm when excited with 760 nm (two-photon) [11], as we have observed.

In addition to using spectral properties, we add to previous fluorescence lifetime approaches studying the composition of cells. The better fit of the bi- and triexponental over the monoexponential decay model in our study suggested—like the spectral analysis—that two or more autofluorescence components were present in bedbug sperm. The lifetime rather suddenly shifted from mean values of *τ*_m_ ≈ 1700 ps at excitation wavelengths of less than 780 nm to much shorter lifetimes when excited with longer wavelengths. The interim lifetime value found at excitations of 800 nm suggests that the lifetime switch is associated with excitation around this wavelength. This pattern is consistent with a relatively sudden decrease of the NAD(P)H contribution to the total signal at excitations more than 800 nm.

By fitting two-exponential decays, we found four peaks of lifetime distributions: for *τ*_1_ at 180 ps (peak A) and 920 ps (peak B; figure 7*a*) and for *τ*_2_ at 2500 ps (peak C) and at 3800 ps (peak D; figure 7*b*). Peak A is in the range of fluorescent lifetime values for free NAD(P)H (0.2–0.4 ns) [5,41], whereas peaks C and D are in the range of fluorescence lifetimes of protein-bound NAD(P)H and free flavins (averaging 2–3 ns, but reaching up to 4 and 6 ns) [5,41–43]. These suggestions would be consistent with the results of our triexponential fit, where the 3800 ps peak may correspond to free flavin, the 1500 ps peak to bound NAD(P)H and the shortest peak to free NAD(P)H [44].

Flavins and NAD(P)H have an important role in cell metabolism and their relative presence is used to characterize the redox states of cells, including sperm [8,9,40,44]. Variation in mean fluorescence lifetime may, therefore, be used to compare sperm metabolic properties in males and females. We are not aware of previous attempts to do so, except for two studies in insects [9,10].

The autofluorescence lifetime method presented here is also a convenient way to compare optical characteristics of sperm metabolism with those provided by invasive physiological and biochemical methods. Several papers examined in sperm the enzyme activity of ATP metabolism related to either glycolysis or oxidative phosphorylation [38,45–47]. However, a comparison with FLIM has, so far, only been carried out once, in cancer cells [48]. Importantly, FLIM is independent of cell density.

### 4.2. The significance of a female-mediated sperm metabolism

We found greater variation in the fluorescence lifetime distribution in sperm sampled from females compared with that sampled in males (figure 5). This pattern is consistent with the female sperm manipulation hypothesis, but not consistent with the idea that sperm within the female sperm storage organ represents an unaltered subset of the sperm function found in males (figure 1). This result, suggesting metabolic differences between sperm cells stored by the male and the female, complements previous studies [9,10]. One of the studies further showed that the sperm function measured in the female does not mathematically predict the metabolic rate of the same sperm in the male [9]. It is currently, therefore, not clear how females may obtain information about the male genotype (‘good genes’) from the male's sperm function alone. In conjunction with small heritable components of sperm traits observed in other studies [49,50] and the observation that postcopulatory function only explains a small part (<2%) of the total variation in male reproductive success [51], our findings add to the suggestion that sexual selection at the level of the sperm cell (sperm choice or sperm competition *per se*) may have lower evolutionary significance than is currently assumed (see Introduction).

It is impossible with the present data to suggest by which precise mechanism females manipulate sperm. However, given that peak D (putative protein-bound NAD(P)H or free flavin) was present in only half the sperm samples extracted from females, a female interference with sperm metabolism appears possible (see also [26]). Such interference was observed previously when the reduced metabolic rate occurring in sperm extracted from females, compared with that of males, was correlated with a reduced production rate of oxygen radicals [9,10]. Hypothetically, the data presented here may be linked to the possibility that female insects, and perhaps other animals [25,26], interact with sperm cell metabolism in such a way that the concentrations of flavins and flavoproteins are reduced. Flavins and flavoproteins are sources of oxygen radicals [52,53]. Females may also reduce the metabolic rate of sperm, resulting in less protein-bound NAD(P)H, compared with free NAD(P)H. As one theoretical way to reduce sperm metabolism, it has been suggested that females confine sperm at high cell density within the sperm storage organ. This could reduce sperm activity and thereby extend sperm lifespan [20].

## 5. Conclusion

We provide a proof of concept that two-photon FLIM is a suitable method to examine sperm characteristics without the need to stain cells. We have used insect sperm cells as simple models of cellular metabolism *ex vivo* but, given that the insect cuticle possesses a defined autofluorescence, there may be exciting prospects for the development of *in vivo* applications to address further medical as well as evolutionary questions. The spectral and fluorescent lifetime components of sperm cells can be used to infer that sperm undergo metabolic changes when entering the female storage organ. How these changes vary across species may be a fruitful approach to look into explanations of the large diversity of sperm cells.

## Supplementary Material

data Fig2

## Supplementary Material

data Fig4

## Supplementary Material

data Fig6
